# Reducing the environmental impact of nitrous oxide in dentistry: a national quality improvement project

**DOI:** 10.1038/s41415-025-9201-6

**Published:** 2026-01-23

**Authors:** Alexandra Lyne, Sarah Ahmad, Paul Ashley

**Affiliations:** Paediatric Dentistry, Eastman Dental Institute, Rockefeller, 21 University Street, London, WC1E 6DE, United Kingdom

## Abstract

**Background** Nitrous oxide (N_2_O), used in dentistry for inhalation sedation, is a greenhouse gas. Advice on how to mitigate the environmental harm in healthcare settings is not dental-specific.

**Aim** The aim was to quantify the environmental impact of N_2_O used in UK dentistry, and make recommendations to reduce the environmental harm, while maintaining patient care.

**Method** This study followed quality improvement project methodology. Following a pilot at the Eastman Dental Hospital in London, a national protocol was devised and advertised through the British Society of Paediatric Dentistry in 2022. Participating services audited the carbon footprint (measured in kilograms of carbon dioxide equivalent [kg CO_2_e]) of their sedation. Where possible, wastage was estimated.

**Results** In total, 31 dental services across the UK returned data from 891 sedation appointments. The average carbon footprint administered per appointment was 28.62kg CO_2_e (range: 10.74−40.67). Wastage was estimated at 52 out of the 128 participating sites, with an average of 30% at sites using a central piped supply, and 4% at sites using individual cylinders.

**Conclusion** N_2_O used in dentistry has a quantifiable impact on the environment. This study highlighted significant variations in wastage and clinical administration. Recommendations are made for dental services using N_2_O.

## Key points

- Nitrous oxide sedation is a clinically successful form of conscious sedation in the UK, but it does cause quantifiable environmental harm.
- There is wide variation in the volumes of nitrous oxide administered to dental patients, as well as potential wastage in the supply of the gas.
- This paper makes recommendations to address the environmental harm, while maintaining patient benefit.

## Introduction

Inhalation sedation (IS), a form of conscious sedation in dentistry, uses a titrated dose of nitrous oxide (N_2_O) gas. The use of N_2_O in healthcare has a wide margin of safety,^1^ and inhalation sedation is described as a standard technique for dentist-led sedation by the Scottish Dental Clinical Effectiveness Programme^2^ and the Intercollegiate Advisory Committee for Sedation in Dentistry.^3^ It has been found to be clinically successful for dental procedures in children and adults.^4^

Unfortunately, N_2_O is a potent greenhouse gas that contributes to climate change. It is more potent than carbon dioxide (CO_2_) per kilogram, with the latest report from the International Panel on Climate Change finding a global warming potential 273 times greater than CO_2_.^5^ A recent life cycle assessment study found that a single appointment for IS in a dental practice contributed the equivalent of 94.45 kilograms of carbon dioxide equivalent (CO_2_e), with a majority of the carbon footprint from the gas itself, and very little impact from the consumables and equipment.^6^

The National Health Service (NHS) has committed to net zero carbon emissions by 2040, and one of their key strategic areas is the mitigation of anaesthetic gases, including N_2_O.^7^ In anaesthesia, the focus is on decommissioning wasteful manifold systems, as well as alternatives to N_2_O, such as total intravenous anaesthesia.^8^^,^^9^

In 2025, a toolkit for reducing waste N_2_O acknowledged that N_2_O mitigation is relevant to multiple disciplinaries in the NHS, including dentistry.^10^ The recommendations are mostly based on reducing the waste in central manifold supplies, which will not apply to all dental services.

The way in which dentistry uses N_2_O differs from that in medical disciplines, and as such will need a tailored solution to the environmental issue. There are certain patient groups for which suitable alternatives are not available or not practical. The way in which N_2_O is administered by clinicians differs from anaesthetics and maternity. Each dental service will have unique combination of patient case mix, N_2_O supply and scavenging set up. It is important that N_2_O in dentistry is properly analysed before making any decisions on how to best tackle the environmental burden.

The aim of this quality improvement project (QIP) was to quantify the carbon footprint of N_2_O gas used in dental services throughout the United Kingdom (UK), and make recommendations to reduce the environmental harm, while maintaining patient benefit.

## Materials and methods

The QIP methodology was piloted at the Eastman Dental Hospital in London, UK, with an initial cycle in October 2022, and subsequent cycles in February, June, and October 2023. Compared to the first cycle, there was a 20% reduction in clinically administered volumes of N_2_O following the local action plan.^11^

The national QIP protocol was based on this local protocol, and peer reviewed by the Quality Improvement and Research Committee of the British Society of Paediatric Dentistry (BSPD) in June 2023.

Recruitment to the national QIP was voluntary and advertised via email to the BSPD membership in September 2023. Members expressing interest via email were sent a project pack containing the local protocol and data collection tools. Participants returning results were supported by the project lead (AL) in data analysis and forming an appropriate action plan for their service.

Participating dental services were asked to collect baseline data on their N_2_O supply (cylinders versus manifold), which specialties used the supply, and what alternative forms of conscious sedation were offered in the service.

Participants selected a week for data collection. Services with a less frequent sedation service (less than ten episodes of inhalation sedation per week) were asked to increase the period of data collection, for example two weeks or one month.

During the data collection period, the following data were collected at each site, for every episode of inhalation sedation used by any dental specialty:

- Patient demographics – age, sex, American Society of Anaesthesiologists physical status classification (ASA grade)
- Sedation parameters – total flow rate (litres per minute [LPM]), maximum N_2_O concentration (expressed as a percentage), and length of time of N_2_O administration (minutes)
- Procedural information – dental procedure and success. Patient cooperation was not recorded, only the success of the planned dental procedure under inhalation sedation.

Options were given to collect the above information, retrospectively or prospectively, depending on whether the above data were routinely documented in patient notes.

### Data analysis

Microsoft Excel was used for data analysis and summary statistics.

The total volume of N_2_O administered for each episode of sedation was calculated by multiplying the flow rate, maximum N_2_O titration, and length of administration. For example, if the initial flow rate was set as 5 LPM with a maximum titration of 30% N_2_O, given for 20 minutes, the calculation for the volume of N_2_O delivered would be 5 × 0.3 × 20 = 30 L. Any initial increments of N_2_O were not included as they had minimal impact on the overall volume. To convert the volume of N_2_O to a carbon footprint, the N_2_O volume was converted to weight in kilograms (assuming 1 litre of N_2_O weighs 0.001984467 kg),^12^ and multiplied by a global warming potential of 265 (the most recent figure available at the time).^5^ This produced a carbon footprint in kilograms of CO_2_ equivalent (kg CO_2_e). In the example given above, 30 L of N_2_O is equivalent to 15.77 kg CO_2_e.

Where cylinder procurement data were available, wastage was estimated by comparing the procured volume of N_2_O gas with the volume administered to patients. Any shortfall was considered wastage and expressed as a percentage.

As an example for a cylinder-service, if the clinic procured ten size E cylinders in one year (equivalent to 346 L per week), and a total of 300 L was administered to patients during the data collection week, then wastage would be 13%.

As an example for a piped manifold service, if the clinic/hospital procured six size G cylinders in one year (equivalent to 1,038 L per week) and a total of 519 L was administered to patients during the data collection week, then wastage would be 50%. Wastage calculations for piped systems were only possible when the supply was not shared by any other medical or surgical specialties (i.e., not used for theatres).

The proportion of patients that could have been eligible for standard technique intravenous sedation was estimated, based on their age (12 years or older) and ASA grade (Grade I or II).^2^^,^^3^

Participating services received their own results, as well as the anonymised national results, in May 2024.

## Results

In total, 62 dental services expressed interest in participating, of which 31 provided data for analysis. This was spread across 128 primary and secondary care sites in the UK. A majority of participating organisations were Community Dental Services (n = 21), followed by hospital services (n = 9), and one high street dental practice. Data were available for a total of 891 episodes of sedation.

### Carbon footprint

The average carbon footprint in a dental service, for one weeks' worth of clinical administration of N_2_O, was 518.25 kg CO_2_e (range: 38.88–1849). Per episode of inhalation sedation, the average was 28.62 kg CO_2_e (range: 10.74−40.67).

### N2O supply and wastage

Of the 128 sites providing inhalation sedation, 84% (n = 108) had a cylinder supply (cylinders attached to the dental sedation machine in each surgery) and 16% (n = 20) had a piped supply (cylinders attached to a central manifold and piped into each dental surgery).

All services used active scavenging via a central scavenging system, mobile scavenging unit, and/or using high-speed suction.

Data were collected for wastage estimations at 42 cylinder sites and 10 piped sites, with an overall average of 9% wastage. On average, there was less wastage in the cylinder group (4%, compared to 30% in the piped group), with a large variation in wastage across both groups (-271% to 77% in the cylinder group, and -87% to 91% in the piped group). Negative wastage estimations were calculated at 15 sites. This occurred when the volume of gas administered to patients was greater than the expected volume available in the cylinders for that time period. This was assumed to be because the period of data collection was unusually ‘busy' compared to a typical week in that service.

### Administration of N2O

**Fig. 1:**
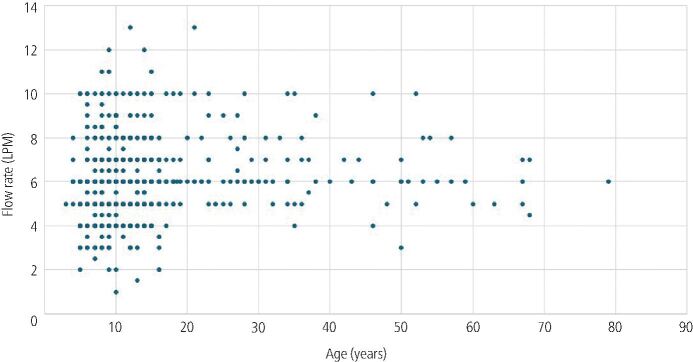
Flow rate (litres per minute) and patient age (years)

The average flow rate was 5.84 LPM, with a range of 1–13 LPM. Figure 1 shows the flow rates used for patients of different ages. There did not appear to be a visual correlation between age and flow rate.

The average titrated dose of N_2_O was 34.5%. All but one titration were within the range of 10–55% N_2_O, with a single outlier at 70% N_2_O. The average length of N_2_O administration was 28 minutes (range: 3–99 minutes).

### Dental procedure

**Fig. 2:**
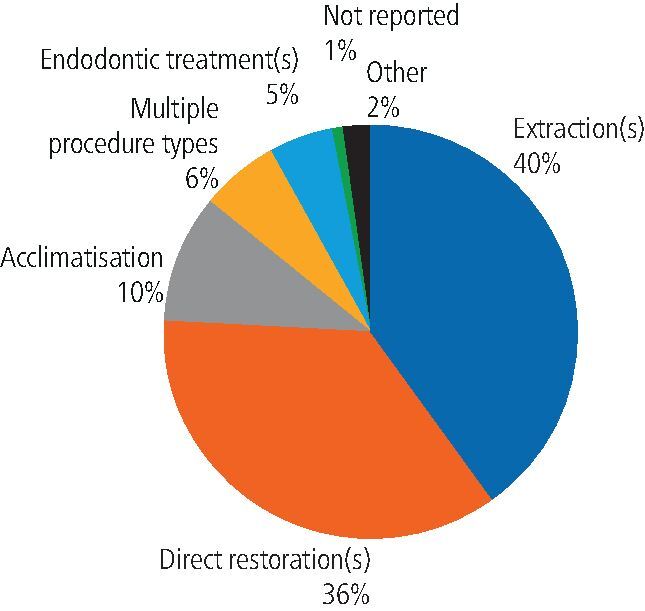
Dental procedures under inhalation sedation

The spread of dental procedures carried out is shown in Figure 2. A majority of the procedures were extractions or restorations.

The overall national success rate for the 891 patients was 92% (this ranged from 74–100% between individual services).

Additionally, 19 out of 31 services reported using IS for acclimatisation procedures only – these patients had simple, non-invasive procedures (e.g., fissure sealants or polishing teeth) to acclimatise or desensitise the patient. The average success rate in services offering routine acclimatisation was 91%, compared to 94% success in services that did not offer acclimatisation visits routinely.

### Patient age and sedation alternatives

Of the 891 episodes of sedation included, 83% were paediatric patients (15 years or younger), and 17% were adult (16 years or older).

The only alternative form of conscious sedation being offered by the participating services was intravenous sedation (IVS) using midazolam. There were 19 services that were able to offer IVS at the time of participation. These services had various age criteria for IVS, for example adults only (n = 11), 12 years and older (n = 6), and 8 years and older (n = 2). The two services offering IVS to age eight and over were doing so with an anaesthetist present, and in line with advanced sedation guidelines. In addition to the age criteria, three of these services restricted what treatments were offered under IVS (i.e., extractions only).

Of the 891 episodes of sedation, 40% of patients may have been suitable for standard IVS technique, based on their age and ASA grade.^2^^,^^3^

## Discussion

This is the first study to quantify the real-life environmental impact of N_2_O in dentistry across different settings in the UK. It supports previous data that the use of inhalation sedation in dentistry contributes to the environmental burden.^6^ Any dental service using N_2_O should be aware of this impact and take steps to reduce it.

The carbon footprint in this QIP is based on the N_2_O gas administered to patients, and does not include the environmental footprint of wasted gas, materials, running the dental surgery, servicing equipment, or patient and staff travel. This QIP was conducted in 2023/24, when the global warming potential of N_2_O was 265.^5^ This has since been updated to 273, so the carbon footprint is likely a slight underestimation.

The high success rate of IS observed in this study aligns with existing literature,^4^ reinforcing the benefit of this form of sedation for dental patients, especially considering alternatives are not universally available in all services, and have more restricted eligibility criteria. A majority of the sedation episodes were for paediatric patients, where N_2_O sedation is the only standard sedation technique available.^2^^,^^3^ Given the benefit to patients, and lack of a like-for-like alternative, the authors support the continued use of N_2_O in dentistry at this time, where it is supplied and administered responsibly.

The study identifies significant variation in the clinical administration of N_2_O across the country, highlighting the relatively unregulated nature of this form of sedation and the need for standardised staff training on administration. As an example, flow rate varied greatly between services and had a greater impact on the overall carbon footprint than the N_2_O titration. A flow rate that is greater than clinically necessary does not hold any additional patient benefit but does increase the volume of N_2_O used, and therefore the carbon footprint and harm to the planet. Flow rate did not appear to relate to patient age, so it is likely that the variation reflects clinician training or preference. Another possibility is that the scavenging volume is too high, meaning that higher flow rates are needed to maintain the volume in the reservoir bag. Scavenging is recommended at 45 LPM in dental sedation guidelines,^2^^,^^3^ but there is no standard about how or when this should be tested. The 45 LPM figure is based on the average lung capacity of an adult, and therefore the authors of this study question whether it should be applied to every patient having dental sedation without further evidence.^13^ The authors advocate for further research into safe scavenging levels, and how to test scavenging levels in daily practice, as this may help to reduce the flow rates needed to inflate a reservoir bag. Alternatively, sedation machines that eliminate the need for a reservoir bag have the potential to reduce the gas volumes administered to patients.

A variety of dental procedures were provided under IS in this study, including acclimatisation procedures in some services. An additional visit to acclimatise the patient to IS results in more N_2_O used for that patient's course of treatment, as well as more visits for that patient to attend. Although acclimatisation aims to increase the success rate of treatment, this study did not observe higher success rates for services using sedation for acclimatisation, compared to those that did not. Given the lack of difference in success, and the additional environmental cost, this study would not support acclimatisation under sedation as routine. The decision on whether to offer an acclimatisation visit under sedation should be considered on an individual patient basis.

Wastage refers to the proportion of N_2_O gas that is not used for patient care. Wastage estimations in this study were based on short data collection periods and relied on patient notes, and so this is considered an estimation rather than formal calculation of wastage. The method of wastage estimation differs from studies in anaesthetics, where the gas volume is measured by the anaesthetic machine, so comparison of the wastage figures in this study to other healthcare settings is not appropriate. Although the average wastage was greater in sites with a central piped supply, there was huge variation in wastage in both cylinder and piped supplies. There are no set standards on what an acceptable level of wastage is. The authors suggest that wastage is monitored in every service providing sedation, regardless of cylinder or manifold supply, and steps are taken to address any potential cause of wastage. During this study, services with ‘high' wastage (greater than 25%) were asked to investigate stock control (for example, were unused cylinders left to expire or stolen, were cylinders changed too early), and faulty equipment (for example, piping leaks and faulty cylinder gauges). Based on the national average volume of N_2_O per episode of sedation, one size E cylinder should last for approximately 32 episodes of IS, if there is minimal wastage.

The main limitation of this QIP was the short data collection periods, meaning that data may not be representative of normal sedation activity throughout the year. Furthermore, the CO_2_e calculation relied on accurate recording of flow rate, time, and titrated dose. Flow rate and titrated dose can vary throughout the sedation procedure, and titrations are typically increased slowly at the start of the procedure. Ideally, the sedation machine would include a metre reading that identifies exactly how much gas has passed through it. This is a feature of anaesthetic machines and would make wastage estimations comparable to anaesthetics, more accurate, and more practical to routinely monitor.

This QIP was conducted on a voluntary basis with no incentive for participation. The QIP was advertised to BSPD members, and so participants may be over-representative of paediatric dentistry, and under-representative of special care dentistry and oral surgery services. Dental practices were also under-represented.

Table 1 outlines some simple recommendations to dental services that use N_2_O, alongside some potential actions or quality improvement activity to reduce the environmental impact of the N_2_O, while maintaining patient care. Recommendations would vary between sites depending on their service, how N_2_O is clinically administered and supplied, and how much is wasted. Ideally, a toolkit would be produced with a step-by-step guide on N_2_O reduction, which is currently in development by the authors.

**Table 1:** Recommendations to dental services providing inhalation sedation

**Recommendation**	**Potential action(s)**
The service identifies areas for N_2_O reduction through quality improvement activity	Undertake audit of N_2_O clinical administration and estimate wastage
Where appropriate to the patient, consider alternatives to N_2_O sedation	Audit demographics of patients having N_2_O sedation, and what alternatives the service can offer Consider how to increase access to alternatives, such as IVS, if a large cohort of patients having N_2_O would be suitable for standard technique IVS
Estimate wastage of N_2_O and take steps to investigate wastage over 20%	Estimate wastage by comparing the procured volume against the volume delivered to patients during clinical audit Investigate and take actions to address any potential causes of waste N_2_O. For example: faulty equipment and leaks, poor stock control (allowing cylinders to expire), user error (changing cylinders before they are empty)
Ensure there is a strong clinical benefit to the use of N_2_O within the service	Audit success rate of N_2_O sedation Consider whether acclimatisation visits are offered, and whether they increase success rate for particular patient groups
Flow rates are kept as low as clinically necessary	Record and audit flow rates Ongoing education of the dental sedation team on appropriate use of flow rates and inflation of the reservoir bag Consider investigating strength of scavenging if issues with inflation of the reservoir bag at 6 litres per minute
The titration of N_2_O is kept as low as clinically necessary for each stage of the dental procedure and is turned ‘off' (e.g., back to 100% oxygen) as soon as the anxiety-provoking part of the procedure is finished	Record and audit N_2_O titration and length of administration, and dental procedure Ongoing education of sedation trained clinicians and nurses, including on using non-pharmacological behaviour management alongside N_2_O Before starting an episode of sedation, there is a pre-operative briefing between the operating clinician and nurse, including discussing whether it would be appropriate to turn N_2_O titration down or ‘off' at a certain stage. This will be individualised for the patient and procedure
IVS, intravenous sedation; N_2_O, nitrous oxide	

In an ideal world, the clinical need for N_2_O in dentistry would be eliminated by a suitable alternative inhalation agent. Further research is needed into potential alternatives, such as methoxyflurane (Penthrox),^14^ and consideration of whether to include it in dental conscious sedation guidelines. Currently, Penthrox is licenced for adults only in the UK, and so would not apply to a majority of N_2_O sedation in dentistry, which is done on children and young people.^15^ Alternatively, the safety and practicality of providing alternative forms of sedation in dental settings (such as midazolam given orally, nasally, or intravenously) should be considered at each guideline update, so that access to these alternatives can be improved, and our reliance on N_2_O reduced.

Finally, industry will play a role in reducing N_2_O in dentistry. As flow rates had the biggest impact on the overall carbon footprint, any innovations that reduce or eliminate the flow rate would have the greatest impact on the carbon footprint. Ideally, all services would have a direct cylinder supply, with two cylinders attached to the mixer via an automated gauge that allows one cylinder to run fully empty, and then switches automatically to the back-up cylinder. Furthermore, it would be ideal if all sedation machines included a ‘metre reading' of how much N_2_O has passed through the machine, as this would make wastage estimations simpler and in line with methodology used in anaesthesia.^8^^,^^9^

Capture-and-destruction technology has been touted as a way to mitigate the environmental impact of N_2_O in healthcare settings, such as maternity.^16^ It is important to note that although this technology has good evidence for the capture of N_2_O molecules (i.e., scavenging and the occupational health benefit), the authors are not yet aware of any real-world data on its effectiveness of destruction of the N_2_O molecules (i.e., the environmental benefit). Additional research is needed into this capture-and-destruction technology in dental settings, including the costs and benefits (both financial and environmental). In line with the latest guidance, until there is further evidence, the authors would recommend that dental services focus on wastage and clinical administration before considering this technology.^10^

## Conclusion

N_2_O used in dentistry has a detrimental impact on the environment. There is a wide variation nationally on how this gas is administered and supplied. It is the responsibility of all dental clinicians who administer inhalation sedation to take steps to reduce the carbon footprint, while maintaining the patient benefit. The authors advocate for professional bodies to consider the environmental impact of N_2_O in all future dental sedation guidelines.

## Data Availability

The data that support the findings of this study are available from the corresponding author upon reasonable request.
